# Unravelling typology of family life when a parent has heart disease: A qualitative study of families with adolescents

**DOI:** 10.1016/j.ijnsa.2025.100324

**Published:** 2025-04-08

**Authors:** Matilda Holmbom, Hanna Grundström, Frida Andréasson, Camilla Rotvig, Hege Andersen, Camilla Bernild, Tone Merete Norekvål, Selina Kikkenborg Berg, Anna Strömberg

**Affiliations:** a Department of Health, Medicine and Caring Sciences, Linköping University, Sweden; b Department of Obstetrics and Gynecology in Norrkoping, and Department of Biomedical and Clinical Sciences, Linköping University, Linköping, Sweden; c Department of Social Work, Linnaeus University, Kalmar, Sweden; d The Heart Center, Rigshospitalet, Copenhagen University Hospital, Copenhagen, Denmark; e Haukeland University Hospital, Bergen, Norway; f Department of Health and Caring Sciences, Western Norway University of Applied Sciences, Norway; g Department of Clinical Science, University of Bergen, Bergen, Norway; h Department of Cardiology, Linköping University, Sweden

**Keywords:** Heart disease, Adolescent, Parenting, Qualitative research, Coping strategies, Support, Family dynamics, Illness adaptation, Typology development

## Abstract

**Background:**

When a parent is living with heart disease, it impacts the entire family. To fully understand the effect, the perspectives of all family members need be analysed together as a unit.

**Objective:**

To identify what characterises family life and relationships in families with adolescents, with a parent living with heart disease.

**Design:**

Qualitative study with an inductive approach.

**Setting:**

In three Scandinavian countries between 2019 and 2022.

**Participants:**

A total of 28 families with 83 family members, from three university hospitals were included. Inclusion criteria were families with a parent living with any type of heart disease, within six months and up to five years since diagnosis and having one or more adolescents living at home.

**Methods:**

Data was collected through semi-structured individual interviews. Reflexive thematic analysis was used to identify patterns within families. This was followed by an ideal-type analysis, which resulted in a typology defining aspects of family experiences and responses of living with heart disease.

**Results:**

A typology was developed describing four different family responses to heart disease: resilient, fragile, overwhelmed, and resigned. A family with a resilient response exhibits a collective approach, fostering solidarity and adaptability as they manage heart disease. A family with a fragile response shares a sense of belonging among family members, but struggles with concurrent stressors, navigating challenges individually without external support. A family with an overwhelmed response experience breakdowns in communication and helplessness in managing heart disease alongside various demands. A family with a resigned response relies on individual strategies leading to challenges for them to interact and understand each other.

**Conclusions:**

Families affected by heart disease handle their new life circumstances in various ways. Strong family cohesion and supporting networks emerged as crucial elements in helping families cope with the multifaceted challenges associated with living with heart disease.

## Abstract (author highlights)

• Chronic diseases significantly impact family dynamics, often altering structure, routines, and redistributing responsibilities to meet the care needs of the person being ill.
• Managing chronic illnesses within a family is complex; the disease can strain relationships due to its challenges, but it also offers an opportunity to strengthen bonds through support and resilience.
• This study identified four family responses: Resilient, fragile, overwhelmed, and resigned, illustrating how family life and relationships can be described using a typology.
• The typology illustrates how family cohesion and external supporting networks influence experiences of coping with heart disease, with the family responses placed along a continuum on these two factors.
• Identifying different family responses may offer a heuristic tool to understand dynamics in families with a parent living with heart disease.

## 1 Background

Living with chronic illness is not just an individual struggle. It disrupts the entire family and resonates to all family members (Meiers et al., 2020). Family members often struggle with concerns about the well-being and prognosis of the person who is ill. In return, the person who is ill may fear burdening the family with their condition. These dynamics often require significant adjustments within the family structure, as roles are redefined, and responsibilities redistributed (Pesantes et al., 2020). The person who is ill needs increased practical and emotional assistance compared to prior to the illness. This adaptation entails navigating through a myriad of emotions, from having to handle limitations to striving to retain hope and see possibilities (Årestedt et al., 2014). The overall experience of the ill person is further complicated by the many feelings of losses, for instance loss of physical abilities or independence, at various stages of the illness trajectory. This can cause emotional stress, sorrow, and feelings of vulnerability (Eggenberger et al., 2011). The inability to fulfil one's primary roles may lead to feelings of frustration, self-blame, and guilt among family members (Pesantes et al., 2020). When a parent suffers from illness, it leads to direct impact especially on minor children living at home, who are dependent on their parents. They are therefore a vulnerable group and are at an increased risk of suffering negative consequences, such as anxiety and depression (Holm et al., 2023). Adolescents facing parental heart disease describe a disturbance in their life path, requiring them to re-evaluate their identity to adapt to the changes in their life (Rotvig et al., 2024). The lives of spouses who are both parents and have partners living with heart disease are significantly impacted. The spouses face both increased daily responsibilities and parenting duties due to their partner's condition (Holmbom et al., 2024). Despite these challenges, the family must strive to adapt and maintain their functionality. This adaptation relies on the ability to effectively manage the stressors associated with chronic illness (Treder-Rochna, 2020), and involves finding a balance between addressing the illness and preserving a sense of normality. This requires reshaping communication patterns, redefining roles, and promoting understanding and acceptance within the family (Årestedt et al., 2014).

It is important to be aware of the complexity of family dynamics in the context of chronic illness. By understanding these experiences, support can be designed that promote family well-being and resilience (Meiers et al., 2020). Previous research has largely focused on individual experiences, with less emphasis on the challenges families with adolescents’ encounter. Balancing various aspects of life is often more challenging when heart disease strikes at a younger age, particularly when family responsibilities are involved (Ivarsson et al., 2019; Nicholas Dionne-Odom et al., 2017). To fully understand the influence on family life of families living with heart disease, it is important to integrate the perspectives of each family member and analyse the dynamics of their relationships as a unit. In our context, we focus on heart disease, marked by unpredictability and sudden events that can heighten stress, strain family relationships, and disrupt daily life (McHorney et al., 2021). Addressing this knowledge gap can enable more precise and effective support measures for families navigating a new life situation. Therefore, the aim of this study was to identify what characterises family life and relationships in families with adolescents, with a parent living with heart disease.

## 2 Methods

### 2.1 Design

This was a qualitative study, conducted in the three Scandinavian countries Sweden, Denmark and Norway between 2019 and 2022. A qualitative approach was chosen to explore family life and relationships in depth. Semi-structured interviews were selected to allow participants to share their experiences in a flexible and open way (Braun and Clarke, 2021). In this study, we conceptualise "family life" as encompassing the collective experiences, interactions, and dynamics within a family unit. This includes examining how family members communicate, support each other and deal with external support, share responsibilities, and navigate challenges together. Similarly, we define "relationships" as the connections, interactions, and bonds between individuals, characterised by dynamics such as emotional intimacy, communication patterns, mutual support, and shared experiences. In this study, we specifically focus on exploring these connections within the family context as it is the family unit that is being studied.

### 2.2 Sampling and settings

We used purposive sampling (Moser and Korstjens, 2018) to recruit participants meeting specific inclusion criteria. The inclusion criteria were families with a parent living with any type of heart disease, within six months and up to five years since diagnosis and having one or more adolescents living at home. Additionally, participants needed to be able to communicate in the interviewer´s native language. Recruitment was conducted at cardiology departments in one university hospital in each country. All patients who met the inclusion criteria and were identified during hospital admission received oral information in person from a nurse, supplemented with a written information letter. Patients identified outside the hospital setting were contacted by telephone and sent written information by post. One week later, patients were contacted by phone to assess interest in participation. After obtaining patient consent, other family members were approached, and written informed consent was obtained from all. We sought diversity in family structure, socioeconomic background, and heart disease severity by recruiting from various hospitals and settings, ensuring a broad range of experiences. Two additional families were approached: the first declined participation due to an adolescent's discomfort, while the second encountered language barriers.

The interviews took place either at home or in hospitals (*n* = 26), and during the Covid-19 pandemic they were conducted over Zoom (*n* = 25) or by phone (*n* = 32). The locations were chosen to ensure confidentiality and privacy by being situated away from the participants' families. In total, 28 families were recruited, comprising eight families (27 participants) from Sweden, ten families (26 participants) from Denmark, and ten families (30 participants) from Norway. The families had one to three children, with a median of two children. Teenagers aged 13–19 were included. The final sample size was determined using the concept of "information power," where no new insights emerged from the interviews, ensuring the sample provided sufficient relevant data for the study's aim (Malterud et al., 2016).

### 2.3 Study participants

The study involved 28 families, including 83 participants in total: adolescents, healthy parents, and ill parents Table 1.

**Table 1:** Demographics and clinical characteristics of participants.

**Age, years, median, (range)**	
Adolescent	16 (13–19)
Healthy parent	47 (40–56)
Ill parent	48 (40–63)
**Gender of, n ( %)**	
Adolescent	Male 15 (45 %), Female 18 (55 %)
Healthy parent	Male 6 (27 %), Female 16 (73 %)
Ill parent	Male 21 (75 %), Female 7 (25 %)
**Heart diseases, n ( %)**	
Ischemic heart disease	10 (36 %)
Heart failure	6 (22 %)
Arrhythmia	5 (18 %)
Heart valve disease	4 (14 %)
Cardiac arrest	3 (10 %)
**Biological parent´s civil status, n ( %)**	
Married/Co-habitants	21 (75 %)
Divorced	7 (25 %)

### 2.4 Data collection

Individual interviews were conducted by four different researchers in their respective countries and languages. All interviews, with 83 family members in total, were recorded and transcribed. Quotes were translated into English by the interviewer, with consensus reached within the research team. All team members are fluent in both the source language and English. The three Scandinavian countries share similar cultures and languages, allowing to understand each other in their native languages, both spoken and written. The interviewers were one social worker and the rest nurses. All were qualitative researchers but not involved in care of the patient and previously unknown to the participants. A semi-structured interview guide was developed, drawing inspiration from The Family System Illness Model, which conceptualises the family as an interconnected system adapting to illness through changes in roles and dynamics (Rolland, 2018). The model served only as a source of inspiration, as this study followed an inductive approach. The interview guide focused on areas related to the illness, the family as a system, the adolescents, and interactions with health services and professionals. The interview guide is available as supplementary material (Supplementary File 1). The interviews lasted between 20 and 97 minutes, with a median of 38 minutes. Data were stored securely on the research group's servers to ensure confidentiality and compliance with data protection regulations.

### 2.5 Data analysis

Data were analysed using reflexive thematic analysis, which offers flexibility allowing for the identification of patterns in meaning across the dataset to identify themes (Braun and Clarke, 2021, 2006). The researcher plays an essential role in the reflexive analytical process, with reflexivity serving as a key component of the research (Joy et al., 2023). An inductive approach (Braun and Clarke, 2019) was employed to openly explore new perspectives on families' experiences of living with a parent with heart disease. The family was considered as a unit, with the collective responses during the interviews serving as the unit of analysis, rather than individual perspectives. The transcripts were uploaded into NVivo and coded on a family-by-family basis. Through repeated readings of the transcripts, key patterns were identified and coded for relevance. Building on the reflexive thematic analysis, an ideal-type analysis was undertaken to explore deeper, more implicit meanings. This process moved beyond description to construct a typology that reflected the family's unique responses and experiences (Stapley et al., 2022). Two important factors influencing family managing were identified in the data: family cohesion and external support networks. These factors were selected because of their significant influence on how families responded to and managed heart disease. By focusing on these two factors, the typology captures the complex interplay between internal family dynamics and external supportive networks in shaping family responses.

Reflective notes were taken when reading the transcripts. The transcripts and notes were revisited repeatedly throughout the analysis process to confirm that the typology was firmly grounded in the data, and to ensure credibility, breadth, and alignment with the families’ perspectives. This process was crucial to verify that the typology provided an authentic and comprehensive picture of the family's unique circumstances and perspectives. This process of thematic analysis and typology development facilitated a deep understanding of family's experiences of living with a parent with heart disease. Furthermore, it contributed to identifying different ways in which families cope and adapt to a new life situation.

Due to the involvement of multiple researchers, the team first met to collectively review several interviews and ensure consistency in the analysis. Each researcher then analysed their respective datasets (MH, FA, CR, HA). Throughout the process, coders performed reliability checks by reviewing and comparing codes for consistency. The team reconvened at the end of the analysis to reach consensus on the findings. For the secondary ideal-type analysis and the development of the typology, PhD student MH reviewed all data and performed the analysis, which afterwards was discussed with the other researchers. Sensitive data were pseudonymised, with only pseudonymised quotes included in the analysis.

### 2.6 Rigor and reflexivity

The quality criteria for qualitative research involve credibility, transferability, dependability and confirmability (Korstjens and Moser, 2018). Lincoln and Guba proposed authenticity as a set of criteria, emphasizing the importance of aligning findings with participants' lived experiences (Lincoln and Egon, 1985; Patton, 2015). As the study was conducted in collaboration with research teams in Scandinavia, a larger group of participants could be included, enhancing transferability, credibility, and overall authenticity. Frequent meetings and close dialogue within the research team were vital to reaching consensus and ensuring confirmability (Lincoln and Egon, 1985). Interviews were conducted individually to minimise external influence and create a safe environment conducive to participant openness, ensuring the credibility and reliability of the collected data (Patton, 2015). Interviews were conducted in participants' native languages to maintain high quality and dependability (Lincoln and Egon, 1985).

Reflexivity has been cited as a core characteristic of qualitative research and fosters an open and honest narrative (Creswell, 2013). Despite possessing a general understanding of heart disease, the research team's preconceived notions were broad and not specifically tailored to the study's participants. We approached the study with openness when analysing interview data to inform our findings (Pannucci and Wilkins, 2010). Grounded in a constructivist epistemological perspective, we recognise that knowledge is co-constructed between researchers and participants, shaping the findings through interpretation (Patton, 2015; Kiger and Varpio, 2020). Through analysis and reporting, efforts were made to consider the data from various perspectives to improve reflexivity. Presenting a full outline of the data collection and analysis procedure ensures consistency with the selected method, thereby supporting confirmability and trustworthiness (Korstjens and Moser, 2018).

### 2.7 Ethical considerations

This study conforms with the principles in the Declaration of Helsinki ([Bibr bib0002][Bibr bib0002]). The study was initiated after receiving ethical approval from the ethical authority in the respective countries: The Danish Data Protection Agency (P-2019–351), The Swedish Ethical Review Authority (Dnr. 2019–05,310, 2021–02,477), and the Norwegian Regional Ethics Committee for Medical and Health Research (REK 2020/86,482).

## 3 Results

**Fig. 1:**
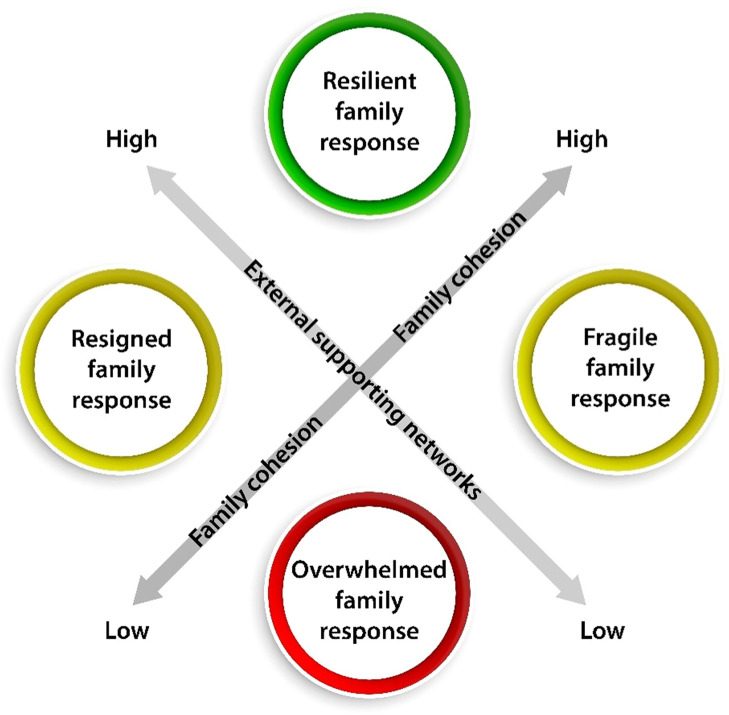
The typology representing four family responses, included 1) resilient, 2) fragile, 3) overwhelmed, and 4) the resigned family response, based on family cohesion and external supporting networks.

The ideal-type analysis revealed a typology consisting of four different family responses, serving as reference points to illustrate the diverse ways families manage heart disease. The family responses included: 1) Resilient, 2) Fragile, 3) Overwhelmed, and 4) Resigned (Fig. 1). This typology was grounded in two central elements identified as crucial in the data: a) family cohesion and b) external supporting networks. Importantly, this typology is not intended as a rigid framework but rather as a heuristic tool to increase our understanding of family responses at a specific point of time (Stapley et al., 2022).

**a) Family cohesion**

Within a typology, a family displays unique behaviours and varying levels of adaptation and resilience, influenced by their specific circumstances, relationships, and resources. This dynamic spectrum may evolve over time and across different phases of the illness trajectory, enabling families to move between different responses. In this study, we use "Family cohesion" to describe how well family members collaborate internally. Cohesion indicates the strength of unity and belonging within the family, with high cohesion showing strong connections and teamwork. This concept reflects how well family members interact, express themselves, and understand each other's needs and perspectives.

**b) External supporting networks**

When we refer to "External support networks," we mean external resources or networks available to individuals or groups, and whether families actively seek and utilise them. These networks include formal services and organisations, such as support groups, healthcare professionals, and social services. Additionally, informal contacts, such as colleagues, neighbours, and friends, play an important role in providing support, thus involving both formal and informal networks.

### 3.1 Family responses

**1) Resilient family response**

A family with resilient response is perceived as a unit sharing the common experience of heart disease in the family that has affected their lives. Despite the challenges posed by the illness, it strengthens their relationships through cooperation and mutual understanding. They confront the disease and its consequences collectively, exhibiting a high degree of adaptability and resilience in managing its impact. Honest and open communication within the family is viewed as very important and natural. They are open to communication and share a common understanding and acceptance of the new life situation brought about by heart disease, which has strengthened their relationships. They have succeeded in using the disease's impact as an opportunity to grow closer and develop new communication patterns and collaboration strategies. A family with resilient response of strategies for coping with heart disease include active communication, mutual understanding, and seeking and accepting help from their networks.

*“The positive thing that has come out of this is that we have become much more united and that is what matters: family, family, family. Family has rooted a different meaning because we know we can lose it”. (Norwegian healthy parent family 2)*

*"We are a very close family that can talk about everything, and we are careful to talk about everything. Because we always talk openly about lots of things, the children are a bit like that… in our family there is more, all feelings are welcome”. (Swedish healthy parent family 1)*

Support resources for a family with resilient response include tailored support groups, healthcare services, counselling, educational materials, and connections with others facing similar challenges. The family demonstrate a high capacity for actively seeking support and readily accepting offered help. They can assess their needs for support and exhibit no reluctance in seeking external support when necessary, notwithstanding their robust intra-familial support structure. Their openness to external support contributes to their ability to address challenges from various perspectives and expertise. Thereby further enhancing their capacity to effectively navigate the impact of heart disease.

*“I have also had supportive hands around me. I have had good colleagues who got in touch and asked how things were going. I didn't feel like I was all alone in it either, so while I was supporting, there were others who supported me”. (Norwegian healthy parent family 9)*

*“There are people, friends and peers, school and everything has really helped. Neighbours have come over and asked us how he's doing and brought us flowers and said they're thinking of us. They know that it's not just dad who's going through this”. (Norwegian adolescent family 5).*

A family with resilient response has collectively agreed to accept the new life conditions. By navigating tough situations together, family members forge bonds that extend beyond what is commonly considered "normal" family ties. A family with this kind of response already had strong relationships before the onset of the illness, which have become even stronger, undergoing experiences that other families do not or cannot comprehend. Even though they are affected by the illness and face difficulties, they choose to find common strategies and collective solutions. A family with resilient response is characterised by a deep sense of connection and alignment between its members. They demonstrate an ability to recover and grow through adversity together as a unit. They actively confront the challenges posed by the disease and use it as an opportunity for personal and familial growth. Their positive outlook and problem-solving abilities make them strong and resilient in managing heart disease.

**2) Fragile family response**

A family with fragile response poses high family cohesion, indicating emotional bonds and a shared sense of belonging among family members. However, despite this cohesion, they are considered as fragile in their ability to navigate the challenges posed by heart disease. Such as frequent medical appointments, managing complex medication regimes, emotional stress, financial strain, and changes in family roles and responsibilities. Minor stressors or changes have the potential to unsettle their stability, triggering intense emotions and fostering uncertainty about the future. Thereby causing significant worry. Moreover, the heart is regarded as the vital organ for sustaining life, and any complications associated with it are interpreted as life-threatening.

*“I take extra care of my dad; I don't like him to be home alone. I'm afraid it'll happen again, dad isn't completely well yet, it has affected him. It's a bit tough to see. I'm also afraid of getting sick myself knowing this is hereditary.” (Norwegian adolescent family 2)*

Additionally, alongside managing heart disease, a family with fragile response contends with various other life challenges, such as parenting adolescents and concurrent illnesses, which further complicates their coping mechanisms. The presence of diverse needs and expressions among family members can complicate mutual understanding, occasionally leading to feelings of disappointment and guilt within the family.

*“We mustn't forget the age of him [the son 16 years], I'm sure he'd rather be with friends than shuttled back and forth to the hospital. That's what I was thinking myself, I was just thinking that someone else was confirming that what you're thinking is right”. (Norwegian healthy parent family 10)*

*“I'm supposed to be the support and the safe spot, and it feels like sometimes my boys have had to be my support instead and it's become backwards and that's not how it should be, really so I haven't liked it so much. I'm a bit sad that it's become like that because I'm supposed to be their stability and not the other way around”. (Swedish ill parent family 5)*

Despite encountering significant impact, a family with fragile response exhibit hesitation towards seeking or receiving support. Rather than reaching out for help, they often prefer to confront their obstacles autonomously, striving to navigate the terrain of heart disease without external involvement. This disposition towards self-reliance may stem from a variety of factors, including a desire to maintain a sense of control over their circumstances, concerns about burdening others with their struggles, or perhaps a lack of awareness regarding available support resources. Consequently, they shoulder the burden of managing the complexities associated with heart disease largely on their own. They rely on their own interaction and coping mechanisms to handle the myriad challenges they face.

*“There was no one, a person who knew and could talk about everything through such a process and what happens on the family side, what is happening here inside? Because you are torn away from reality, that's what's a bit scary. And then it's up to you and your family to figure out how we get these puzzles to work together. I can imagine that it cost a lot of divorces, because if it is like that, you are not united and trust each other, then it's just a mess. It's because you also come home and are a little child in a way, you can't really do anything. That's at least how it feels, so there are a lot of things going through your head, I think, and then it's also the whole range of emotions.” (Danish ill parent family 3)*

**3) Overwhelmed family response**

A family with overwhelmed response finds themselves overwhelmed by the influence of heart disease, feeling as if they are balancing on a very thin thread. They encounter great demands and pressures from various sources, such as adapt to new routines with medication and managing various symptoms from the disease, while striving to maintain family life. The combination of ongoing responsibilities for everyday life and the altered circumstances brought about by heart disease leave them feeling overwhelmed, struggling to identify resources and strategies to cope with their situation. This may result in feelings of powerlessness and a loss of control over their situation. Family cohesion within this family suffers from overwhelming feelings, which are worsened by pre-diagnosis dynamics. If communication and support were already strained before the diagnosis, these challenges intensify once heart disease enters the family. Family members struggle to express their needs, leading to misunderstandings, frustration, and a sense of isolation. This lack of effective communication deepens the emotional burden and further strains relationships, making it harder for the family to adapt.

*“It was just a lot of focus on mom being sick and all that, and I couldn't just be at home without having to help, and that's what I really wanted to help but… but I couldn't just be, just be myself and just relax or focus on school. I had a lot of focus on my mom being sick, and I had to take care of her, and I had to help her, and that also just took all my focus.” (Danish adolescent family 9)*

*“I think, the boys have, she's had this problem throughout their childhood really, so they're kind of used to her mood going up and down, so they know when okay, we're not going to have this discussion now. I have previously thought that it did not affect them but then yes, then I have noticed even now that they may not always feel so good either and whether it is linked to this or whether it is simply that yes, teenage stuff that makes them not feel good. But as I said, of course it has affected the family, it's inevitable”. (Swedish healthy parent family 5)*

In attempts to cope with the overwhelming feelings of stress and anxiety, a family with overwhelmed response use passive or avoidant strategies to overcome their problems, like avoidance of conflict, minimising problems and denial. Instead of addressing the issues directly, they tend to avoid confrontation and open communication on sensitive and difficult topics. This behavioural pattern may be a way for the family trying to avoid further stress, but it can also prevent them from resolving their problems constructively and create a sense of deadlock.

“*She has, well, I don't really understand why, I've never really understood why, it's a bit worse because she was so young when it happened and I, I was like, "Why are you reacting like that?" "Why are you, are you mad at dad for getting sick?" and it was just, it was kind of scary that she didn't want to sleep over at dads with me, even after he got better. I was a bit annoyed with my sister, I felt sorry for my dad, I couldn't, I didn't like the idea of it, what if she didn't like him anymore? What if she was afraid of him? What if she was angry with him and would never ever talk to him again?” (Danish adolescent family 4)*

A family with overwhelmed response also experiences resistance and unwillingness to seek or receive help or support from external sources. The feeling of hopelessness and isolation can create a barrier to seeking the help they are likely to need, preventing them from receiving the support and guidance that can help them break the paralysing feeling that now dominates. This family face overwhelming challenges due to heart disease, leading to strained communication and avoidance of confrontation. Reluctance to seek external help exacerbates feelings of isolation and hopelessness, hindering their ability to cope effectively.

**4) Resigned family response**

A family with resigned response shows an indifferent approach towards addressing the changes brought about by heart disease. Family members use different strategies to manage the situation, and the lack of family cohesion poses challenges for them to interact and understand each other. Within this family, collaboration is limited with members tending to act independently rather than working together to resolve issues. Each family member may focus on their own coping mechanisms, leading to a fragmented approach where collective problem-solving is rare. It is common for family members to avoid confrontation and instead attempt to sidestep conflicts. This can result in superficial communication and a lack of openness and honesty within the family.

*“No, we haven't talked about it, but I've just felt it myself sometimes that even if I feel, well, often I think he sounds angry, but he's not, it's just his tone and it can also be the other way around and then I've trained myself a lot to just be quiet because I want to avoid everything so that nothing happens.” (Danish adolescent family 2)*

Despite this family's awareness of their differences and challenges, there is little willingness to change. This lack of engagement may strain relationships further, creating emotional and psychological distance where family members are less inclined to share thoughts, feelings, or experiences. This distance result in an environment where each family member feels alone in their struggles, even though they are surrounded by family. The indifferent attitude experienced by a family with resigned response may be the result of a long history of relational and other problems and challenges they have faced in the past. This history has fostered a sense of resignation, indifference, and ignorance towards handling new challenges, including the current heart disease. Furthermore, this family often resort to coping mechanisms such as denial and avoidance when confronting the challenges associated with heart disease. In their quest for stability, they strive to uphold an appearance of normalcy amidst the instability. Doubts may arise within family members regarding their capacity to enact meaningful change, leading to a sense of resignation.

*“I think I would mostly just hide it away, really. I just think it would be so much more comfortable for me; I'm not so good at sitting and talking about that kind of stuff.” (Danish adolescent family 3)*

*“It's not something to think about. It's not something we sit and talk about at the kitchen table at home, nothing like that at all. We, no, no such deep stuff, no we don't talk about that.” (Swedish ill parent family 4)*

Rather than actively confronting challenges within the family, a lack of family cohesion might result in dependence on external individuals in the networks to manage these issues. They may perceive efforts to tackle the challenges as unnecessary and opt to accept their current circumstances.

*“No, not really. I have a sister who is very supportive, she is very close to our children, so [child's name] has spoken to my sister instead.” (Danish healthy parent family 2)*

*“I think she [daughter] thinks it's better to talk to friends than anyone else, because they speak a slightly different language than we do” (Norwegian ill parent family 1).*

This reliance not only undermines the family's sense of autonomy and control over their situation but also maintains a cycle of dependency. Consequently, the family members feel disempowered and unprepared to confront their own challenges, fostering a sense of incompetence and apathy. A family with resigned response adopts an indifferent attitude towards addressing heart disease challenges. Limited and superficial communication and independence among family members hinder collaboration, often leading to superficial interactions.

## 4 Discussion

This study provides empirical support for the existence of diverse responses families adopt to navigate the specific challenges of heart disease and highlights its impact on family dynamics. The developed typology representing four family responses: 1) resilient, 2) fragile, 3) overwhelmed and, 4) resigned, illustrating the various ways in which families manage living with heart disease. This typology underscores the significant role of family cohesion and external supporting networks in shaping the experiences of living with heart disease within the family unit. A chronic disease diagnosis at a younger age is often more unexpected, leaving both the individual and their family unprepared for the life changes (Ivarsson et al., 2019). In particular, we would like to highlight the challenges faced by families in coping with the unpredictability and sudden deterioration caused by heart disease (McHorney et al., 2021). Central to these factors is the ability to adapt to the life situation and communicate effectively about it. The dynamic nature of family responses, as families adapt to their evolving circumstances, further underscores the importance of considering this fluidity in understanding the typology. Families may alter between responses depending on changing circumstances, illustrating the dynamic nature of each family's situation.

Our results reveal varying reactions among families, consistent with findings in other disease contexts like parental cancer (Inhestern et al., 2021). Positive effects of the disease can include increased family closeness, while negative effects encompass fear and anxiety in both contexts. Heart disease also poses unique challenges which may intensify stress for the whole family (Rosenstrøm et al., 2022). Similar to our findings, parents affected by cancer also report difficulties in managing their children's reactions, alongside the dual responsibilities of being both a parent and a patient (Inhestern et al., 2021). This is particularly true in families with adolescents, a phase when family dynamics are shifting, and family communication may face greater challenges (Rotvig et al., 2024). This suggests that reactions and coping mechanisms in crises encompass a wide range of emotions, with conflicting feelings coexisting, as we describe in our typology. Perak et al. (Perak et al., 2024) highlight the importance of communication and external support to maintain family stability when facing serious illness, which aligns with our study. What distinguishes our context is the specific focus on heart disease, which is marked by unpredictability, adding an extra layer of stress and anxiety for both patients and their families (McHorney et al., 2021). This difference influences the types and timing of support needed by families living with heart disease. While there are transferable elements in how families respond to various serious illnesses, our study highlights the unique challenges specific to heart disease and how these challenges influence family coping mechanisms.

Our findings highlight the importance of supporting networks and cohesion within families when facing challenges, aligning with frameworks such as the Family Resilience Theory (McCubbin et al., 2001). There are similarities between this theory and the described resilient family response, in our typology, as both emphasise how families mobilise resources and adapt to stressors to maintain functionality and well-being. A family with resilient response demonstrates high family cohesion and strong external support, enabling them to navigate challenges effectively. They communicate openly, and adapt proactively, maintaining a sense of control over their situation. McCubbin and McCubbin define family resilience as the collective characteristics, dimensions, and capabilities that enable families to address challenges by problem-solving and enhancing the adaptability of family members in crisis situations (McCubbin et al., 2001). Family resilience is influenced by various factors, including the duration of adverse circumstances faced by the family, the life stage at which families encounter challenges or crises, and the sources of internal or external support utilised by families during times of adversity. Conceptually, family resilience can be viewed as both a quality and a process ([Bibr bib0009][Bibr bib0009]). A family with fragile response has high family cohesion but limited external support. While they stand together as a unit, the absence of external resources increases their vulnerability, making it more difficult to manage the emotional and practical demands of heart disease. This further underscores the importance of external support systems in strengthening a family's ability to adapt to adversity, as emphasised by the Family Resilience Theory (McCubbin et al., 2001).

The Typology Model of Family Adjustment and Adaptation emphasises the importance of understanding why some families thrive while others struggle in the face of stressors. The model identifies avoidance, elimination, and assimilation as coping strategies to manage stressors, highlighting the significance of a shared understanding of the situation and available capabilities in determining family responses. However, these strategies are influenced by existing resources or capabilities within the family. The model provides insights into how families navigate stress and crises, offering a spectrum of adaptation from positive to negative outcomes. It identifies various dimensions such as family communication, emotional adjustment, and utilisation of external resources (Fouché, 2008). Our findings illustrate how families within our typology demonstrate these adaptive mechanisms in response to heart disease. A family with resigned response having low family cohesion but access to external support tend to adopt a passive approach, relying more on external systems while showing limited engagement in managing the illness within the family. For a variety of reasons, there is a resistance to change, and instead they choose to accept their situation. This indifference may stem from relational difficulties, personality traits, and the way they communicate, as well as a scepticism toward proactive problem-solving.

A family with overwhelmed response experiences both low family cohesion and low external support. Balancing responsibilities with illness challenges is overwhelming, leading to strained relationships and loss of control. Communication breakdowns and avoidance worsen isolation and helplessness. Despite needing support, they may struggle to seek it, worsening distress. According to Fouché, bonadaptation reflects a minimal discrepancy between demands and capabilities, maintaining family integrity and well-being, while maladaptation represents imbalances or compromises at the cost of family cohesion and individual well-being (Fouché, 2008). This distinction aligns with our typology, as families with resigned and overwhelmed response exhibit characteristics of maladaptation, which refer to imbalances or compromises at the cost of family cohesion and individual well-being (Fouché, 2008). These families demonstrate compromised well-being and limited capacity to adjust to heart disease-related stressors, reinforcing the applicability of the Family Resilience Theory and the Typology Model of Family Adjustment and Adaptation (McCubbin et al., 2001; Fouché, 2008). Our typology, tailored to the context of heart disease, simplifies the complex models presented by McCubbin and Fouché (McCubbin et al., 2001; Fouché, 2008), addressing a specific knowledge gap. Through understanding and predicting family behaviours, typologies aid in navigating stressful events. Thus, our study contributes to the broader discussion on family adjustment and adaptation in the context of heart disease, shedding light on coping strategies and resilience factors essential for family well-being.

By defining and describing different family responses, we can gain a better understanding of the different responses and needs that arise in the context of managing heart disease. The family, as a fundamental institution within society, operates as a dynamic system, often evolving, particularly in response to crisis situations faced by its members ([Bibr bib0009][Bibr bib0009]). Although these family responses are abstract constructs, and families cannot be easily categorised or labelled, nor are they static within a single classification, they serve as heuristic tools for understanding how families cope with illness. Observing these responses helps identify challenges and needs, enabling the development of tailored support and resources. They highlight diverse family dynamics and behavioural patterns in disease management. The typology in this study provides insight into how families navigate heart disease, from resilience to overwhelming, showcasing distinct coping mechanisms and challenges. Our findings align with research indicating that persons with heart disease and their closest family experience better family health and functioning when they receive greater social support. These relationships underscore the importance of a dyadic, family-centered approach to enhance family functioning in heart disease contexts (Shamali et al., 2019). Our study contributes to a clearer understanding that families handle and communicate about their challenges in various ways, necessitating tailored forms of support.

### 4.1 Strengths and limitations

Interviews were conducted using various mediums, which was necessary due to the circumstances of the pandemic. Digital channels provided convenience, saved time, and allowed participants to engage from the comfort of their homes. With a total of 83 participants across 28 families, the diverse sample size offers a robust representation of family experiences. However, the different interview mediums might have influenced the depth and tone of responses, as non-verbal cues and interpersonal dynamics can vary between in-person and digital settings. Despite these potential differences, previous research suggests that digital and face-to-face interviews yield comparable quality in data collection (Krouwel et al., 2019). Conducting individual interviews ensured depth and focus on personal experiences, allowing participants to speak more freely about their feelings and challenges without the influence of other family members (Patton, 2015). Nonetheless, opting for individual interviews instead of family interviews may have limited the understanding of how heart disease affects the entire family unit. While family interviews could have offered a more thorough perspective on the family's collective experiences and interactions, not all families may have been comfortable with group interviews, potentially limiting their willingness to open up and share their thoughts and experiences. When constructing a typology through inductive analysis, there's a risk that the typology may impose the researcher's worldview onto participants' experiences, potentially skewing the interpretation (Patton, 2015). We aimed at being attentive and reflective throughout the process to ensure that the typology accurately represented the reality of the families. While the findings and typology may have broader relevance to families with various chronic illnesses, this potential extension should be approached carefully to prevent overgeneralisation and ensure applicability (Korstjens and Moser, 2018).

Cultural and healthcare system differences may have shaped families' responses to heart disease by influencing support, access to care, and caregiving expectations. As our study is context-specific, caution is needed when applying findings to other settings. Language barriers could have excluded families with migrant background, limiting the diversity of the sample, while families with high distress including relational, economical or psychosocial problems may have been underrepresented, which could have influenced the breadth and transferability of the results. Finally, selection bias may also have limited transferability, as participants who agreed to participate may differ from those who declined, potentially affecting the findings. Future research could examine these external factors further.

### 4.2 Recommendations for further research

Exploring support preferences for each family response could help optimise tailored support. A tool to assess and predict family responses may improve this process. Group interviews could capture diverse perspectives within each family response. It would also be important to investigate whether the typology is applicable to families with younger children, as family dynamics and support needs may differ across age groups. Given the complexity and mobility of family dynamics, future research could include longitudinal studies to examine how families move between different responses and how responses to heart disease evolve.

### 4.3 Implications for policy and practice

The typology can be used to better understand how families respond and cope with heart disease in different ways. These insights are important for many different professionals working with families affected by heart disease, to guide the development and adaptation of family-focused support to better meet the specific needs of each family. For example, a family with overwhelmed response may need a different form of support than a family with resilient response. Tailoring support interventions to the unique needs of families with each response can enhance the likelihood of positive outcomes, thereby improving the well-being of the whole family.

## 5 Conclusions

Our study highlights a diversity among families in adapting to and managing heart disease. Understanding these variations is important to comprehend the complexity of family reactions and coping mechanisms to adapt to a new reality. By considering families as cohesive units, this typology may serve as a valuable heuristic tool to identify and understand the different needs and challenges families face when dealing with heart disease. Family cohesion and the establishment of external supporting networks merge as crucial components to help families navigate the multifaceted challenges when living with heart disease. Helping families to prioritise their needs and guiding them through this transition are fundamental aspects of professional care. This can promote positive care experiences among family caregivers and ultimately help to preserve family well-being.

## Funding sources

This work was supported by the 10.13039/501100000329Novo Nordisk Foundation (grant number NNF180C0034016), and the Kamprad Family Foundation (ref 20210130). The funding sources had no role in the design or conduct of the study.

## Declaration of generative AI in the writing process

During the preparation of this work the first author used ChatGPT - OpenAI in order to improve language and readability. After using this tool, the first author reviewed and edited the content as needed and take full responsibility for the content of the publication.

## Data availability

Data is not available for sharing due to concerns about privacy and confidentiality related to qualitative interview data.

## Declaration of competing interest

The authors declare that they have no known competing financial interests or personal relationships that could have appeared to influence the work reported in this paper.

## CRediT authorship contribution statement

**Matilda Holmbom:** Writing – original draft, Visualization, Validation, Methodology, Investigation, Formal analysis, Data curation. **Hanna Grundström:** Writing – review & editing, Supervision, Methodology, Formal analysis. **Frida Andréasson:** Writing – review & editing, Validation, Supervision, Methodology, Investigation, Formal analysis. **Camilla Rotvig:** Writing – review & editing, Validation, Investigation, Formal analysis. **Hege Andersen:** Writing – review & editing, Validation, Investigation, Formal analysis. **Camilla Bernild:** Writing – review & editing, Validation, Investigation. **Tone Merete Norekvål:** Writing – review & editing, Validation, Resources, Formal analysis. **Selina Kikkenborg Berg:** Writing – review & editing, Validation, Supervision, Resources, Project administration, Funding acquisition, Formal analysis, Conceptualization. **Anna Strömberg:** Writing – review & editing, Validation, Supervision, Resources, Project administration, Methodology, Funding acquisition, Formal analysis, Conceptualization.

## Declaration of competing interest

None.

## Appendix Supplementary materials

Image, application 1
