# Supplementary material for: Unravelling typology of family life when a parent has heart disease: A qualitative study of families with adolescents
Source: Int J Nurs Stud Adv. 2025 Apr 8;8:100324. doi: 10.1016/j.ijnsa.2025.100324 (PMC12018042; doi:10.1016/j.ijnsa.2025.100324)
Supplement: Supplementary file 1 [file mmc1.docx]

**Supplementary File 1: Interview guides**

**Parent with heart disease**

**Introduction:**

- "Can you tell me about yourself and your family?"

**1. The heart disease and its impact on the family:**

- "Can you share how your condition started and what the journey has been like for you?"
- "What changes, if any, have you noticed in your daily life?"
- "How would you describe the way your family has adjusted or responded to your condition?"
- "Have there been any positive moments or experiences during this time?"

**2. Parenting and the child’s experience:**

- "Has your role as a parent changed in any way since you became ill? If so, how?"
- "How would you describe your children’s reactions to your condition?"
- "What impact, if any, do you think your illness has had on your children’s lives?"
- "How have you talked about your illness with your children?"

**3. Support and coping strategies:**

- "What strategies have you found helpful for managing as a family?"
- "What kinds of support, if any, have you received, and how has it helped?"

**4. Health and well-being:**

- "How would you describe the impact of your illness on your overall well-being?"
- "When you think about the future, what comes to your mind?"

**5. Social life and relationships:**

- "How has your free time or leisure activities changed, if at all?"
- "What changes, if any, have you noticed in your family’s social life or relationships?"

**6. Concluding thoughts:**

- "How has it been for you to talk about these topics today?"
- "Is there anything else you’d like to share, or feel is important to mention?"

**Healthy parent**

**Introduction:**

- "Can you tell me about your family and your roles within it?"

**1. Heart disease and the family:**

- "What was it like for you when your partner became ill?"
- "What, if anything, has changed in your family’s daily life?"
- "Have there been any particularly challenging or rewarding moments during this time?"

**2. Parenting:**

- "How would you describe your role as a parent since your partner became ill?"
- "What reactions, if any, have you noticed in your children?"
- "How have you talked to your children about the illness?"
- "What kind of support do you think has been helpful for your children?"

**3. Health and well-being:**

- "How has your partner’s illness affected your health and well-being, if at all?"

**4. Work:**

- "Has your work life changed in any way since your partner became ill?"

**5. Social life and leisure:**

- "What changes, if any, have you noticed in your personal free time?"
- "How would you describe your family’s social life now, compared to before the illness?"

**6. Support:**

- "Have you received any support from healthcare professionals or other sources? If so, what kind of support?"

**7. Concluding thoughts:**

- "How has this conversation been for you?"
- "Is there anything else you’d like to add or discuss?"

**Adolescent**

**Introduction:**

- "Can you tell me a bit about your family and who you live with?"

**1. Parent's heart disease:**

- "What do you remember about when your parent became ill?"
- "How would you describe how things have been for you since then?"

**2. Information and involvement:**

- "What do you know about your parent’s condition?"
- "Is there anything you’ve wanted to understand more about it?"

**3. Practical responsibilities:**

- "How, if at all, has your daily life changed since your parent became ill?"

**4. Family dynamics:**

- "How would you describe your family life now compared to before your parent became ill?"

**5. Health and well-being:**

- "How do you feel about your parent’s illness? How do you handle those feelings?"

**6. School/work:**

- "Has your parent’s illness affected your schoolwork or other activities? If so, how?"

**7. Social life/friends:**

- "What changes, if any, have you noticed in your social life or time with friends?"

**8. Support from healthcare:**

- "Have you or your family received any help or support from healthcare professionals?"

**9. Needs for support:**

- "What kind of support do you think would help you the most?"

**10. Closing question:**

- "How has it felt to talk about these things today?"
- "Is there anything else you’d like to share before we finish?"
